# Ferroelectric Wide‐Bandgap Metal Halide Perovskite Field‐Effect Transistors: Toward Transparent Electronics

**DOI:** 10.1002/advs.202300133

**Published:** 2023-01-26

**Authors:** Jiangnan Xia, Xincan Qiu, Yu Liu, Ping‐An Chen, Jing Guo, Huan Wei, Jiaqi Ding, Haihong Xie, Yawei Lv, Fuxiang Li, Wenwu Li, Lei Liao, Yuanyuan Hu

**Affiliations:** ^1^ Key Laboratory for Micro/Nano Optoelectronic Devices of Ministry of Education School of Physics and Electronics Hunan University Changsha 410082 China; ^2^ Shenzhen Research Institute of Hunan University Shenzhen 518063 China; ^3^ International Science and Technology Innovation Cooperation Base for Advanced Display Technologies of Hunan Province College of Semiconductors (College of Integrated Circuits) Hunan University Changsha 410082 China; ^4^ Shanghai Frontiers Science Research Base of Intelligent Optoelectronics and Perception Institute of Optoelectronics Department of Materials Science Fudan University Shanghai 200433 China

**Keywords:** ferroelectric dielectrics, field‐effect transistors, perovskite semiconductors, solution‐process, transparent electronics

## Abstract

Transparent field‐effect transistors (FETs) are attacking intensive interest for constructing fancy “invisible” electronic products. Presently, the main technology for realizing transparent FETs is based on metal oxide semiconductors, which have wide‐bandgap but generally demand sputtering technique or high‐temperature (>350 °C) solution process for fabrication. Herein, a general device fabrication strategy for metal halide perovskite (MHP) FETs is shown, by which transparent perovskite FETs are successfully obtained using low‐temperature (<150 °C) solution process. This strategy involves the employment of ferroelectric copolymer poly(vinylidene fluoride‐co‐trifluoroethylene) (PVDF‐TrFE) as the dielectric, which conquers the challenging issue of gate‐electric‐field screening effect in MHP FETs. Additionally, an ultra‐thin SnO_2_ is inserted between the source/drain electrodes and MHPs to facilitate electron injection. Consequently, n‐type semi‐transparent MAPbBr_3_ FETs and fully transparent MAPbCl_3_ FETs which can operate well at room temperature with mobility over 10^−3^ cm^2^ V^−1^ s^−1^ and on/off ratio >10^3^ are achieved for the first time. The low‐temperature solution processability of these FETs makes them particularly attractive for applications in low‐cost, large‐area transparent electronics.

## Graphical abstract

A strategy of using ferroelectric dielectrics in metal halide perovskite (MHP) field effect‐transistors (FETs) for effectively addressing the gate‐electric‐field screening issue is proposed. By using this strategy, FETs based on MAPbCl_3_ thin‐films are for the first time realized with low‐temperature (<150 °C) solution process. The devices function well at room temperature and exhibit high transparency.

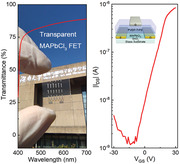

## 1 Introduction

Transparent electronic devices are attracting considerable interest because they could potentially be integrated in glass, in flexible displays, and in smart contact lenses, bringing the products described in science fiction into reality. The key to realize transparent electronics is the employment of wide bandgap (>3.0 eV) semiconductors that absorb little light in the visible range. By far the most widely used semiconductor for transparent electronics is amorphous indium–gallium–zinc–oxide (a‐IGZO), which has attracted much attention for transparent flat‐panel display (FPD) applications like AMOLED since it was first reported by Hosono et al. in Nature, 2004.^[^ ^1^ ^]^ Field‐effect transistors (FETs) based on a‐IGZO show high transparency, decent field‐effect mobility (>10 cm^2^ V^−1^ s^−1^), good uniformity and flexibility, and small subthreshold swing, rendering them promising candidates for the realization of see‐through circuits.^[^ ^2^ ^]^  However, most a‐IGZO films are prepared typically by magnetron sputtering deposition, chemical vapor deposition (CVD), or atomic layer deposition (ALD) processes, which can ensure the high mobility and uniformity of the films, but are not optimal for low‐cost, large‐area electronics. More recently, many studies have demonstrated a‐IGZO FETs by solution deposition processes such as spin‐coating or printing with sol‐gel precursor solutions, yet high‐temperature (>350 °C) annealing processes are generally required for the solution method,^[^ ^2^, ^3^ ^]^ limiting their integration with transparent/flexible plastic substrates. Thus, developing transparent semiconductors and corresponding FETs which can be processed by solution method with low‐temperature process are highly desired.

Metal halide perovskites (MHPs) are an emerging class of semiconductors that have attracted tremendous research efforts because of their excellent photoelectric characteristics.^[^ ^4^ ^]^ The first‐principles calculations show that the intrinsic carrier mobility of MHPs is up to 100–1000 cm^2^ V^−1^  s^−1^,^[^ ^5^ ^]^ suggesting their great application prospects in FETs. Besides, MHPs possess the advantages of solution‐processability and flexibility, which make them very promising for low‐cost, large‐area, and flexible electronics. Moreover, the electrical/optical properties of MHPs can be easily tailored by changing the elemental composition. For instance, the bandgap of methylammonium lead trihalide perovskite (MAPbX_3_, X = I, Br, Cl), the most heavily studied MHPs, can be tuned continuously from 1.6 (MAPbI_3_) to 2.3 eV (MAPbBr_3_) by varying the composition and fractions of the halide element.^[^ ^6^ ^]^ In particular, a wide bandgap of 3.0 eV can be achieved in MAPbCl_3_, which, in combination with the above‐mentioned merits of MHPs, offers us unique opportunities to fabricate transparent devices. However, as well‐documented in literatures, the fabrication of FETs with MAPbX_3_ remains a central challenge, mainly because the devices usually suffer from ion migration in the perovskites, which leads to the screening of gate electric field and thus the misfunction of the device at room temperature.^[^ ^7^ ^]^ Although FETs based on MAPbI_3_ have been shown to operate at room temperature when careful device processing and delicate device engineering techniques were employed,^[^ ^8^ ^]^ transparent FETs made of perovskite semiconductors have not been demonstrated by far.

In this work, by proposing a device fabrication strategy targeting for perovskite FETs, we for the first time demonstrated the availability of top‐gate bottom‐contact (TGBC) FETs based on semi‐transparent MAPbBr_3_ and fully transparent MAPbCl_3_ thin‐films. In specific, ferroelectric copolymer poly(vinylidene fluoride‐co‐trifluoroethylene) (PVDF‐TrFE) is employed as the dielectric, and an ultra‐thin SnO_2_ is used as an interlayer between the substrates and perovskites. We reveal that the combinational employment of the ferroelectric dielectric layer and the SnO_2_ interlayer is indispensable for the achievement of those devices, with the former helping overcoming the gate‐electric‐field screening effect and the latter improving charge injection. The resultant MAPbBr_3_ and MAPbCl_3_ FETs both show electron mobility over 10^−3^ cm^2^ V^−1^ s^−1^ and on/off ratio >10^3^ at room temperature (RT). The low‐temperature (<150 °C) solution processability of these FETs make them particularly attractive for applications in low‐cost, large‐area transparent electronics. Although the performance of these devices remains to be further enhanced, this work provides new opportunities to the realization of transparent electronics.

## 2 Results and Discussion

### 2.1 Preparation and Characterization of the Ferroelectric Dielectric PVDF‐TrFE

PVDF‐TrFE is a commonly used ferroelectric polymer dielectric thanks to its good thermal stability and solution‐processability,^[^ ^9^ ^]^ and it has been frequently employed as dielectric for FETs based on 2D semiconductors and organic semiconductors, yet the exploration of PVDF‐TrFE as dielectric in perovskite FETs is inadequate.^[^ ^10^ ^]^ Previously, Asadi et al. prepared double‐gate perovskite (CsPbBr_3_) FETs with mobility of 10^−5^ cm^2^ V^−1^ s^−1^ and on/off ratio of 10^3^ at room temperature by using PVDF‐TrFE. However, in that work, the ferroelectric PVDF‐TrFE was used as an auxiliary gate to electrostatically fixate the mobile ions rather than a dielectric to induce charge carriers.^[^ ^11^ ^]^

We are motivated to employ PVDF‐TrFE as the dielectric for perovskite FETs by the idea that the dipoles can induce a strong electric field at the semiconductor/dielectric interface, which may overcome the gate‐electric‐filed screening problem caused by ion migration in perovskite semiconductors. Besides, the high transparency of PVDF‐TrFE is also essential for the achievement of transparent FETs.

**Figure 1:**
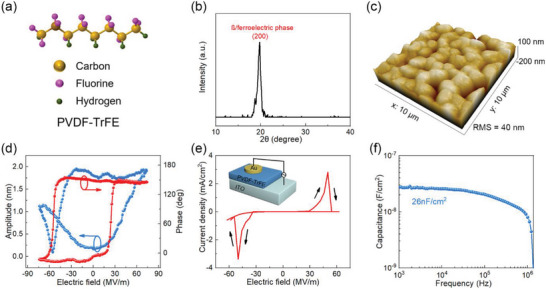
Characterization of PVDF‐TrFE films processed by nBA solvent. a) The chemical structure and b) XRD pattern of PVDF‐TrFE. c) AFM image of PVDF‐TrFE films. d) Hysteresis behavior of (Si/PVDF‐TrFE) observed in the PFM phase and amplitude signals. e) Polarization switching current of the PVDF‐TrFE film with film thickness of 800 nm. The inset shows the structure of the sandwich device for measurements (ITO/PVDF‐TrFE/Au). f) Dependence of capacitance of PVDF‐TrFE film as a function of frequency.

To realize the integration of PVDF‐TrFE with perovskites, the common solvents for PVDF‐TrFE such as dimethyl sulfoxide (DMSO), dimethylformamide (DMF), and 2‐butanone (MEK) cannot be used because they would dissolve perovskites.^[^ ^10^, ^12^ ^]^ To address this issue, we employed n‐butyl Acetate (nBA) as the solvent for PVDF‐TrFE in this study. **Figure** **1**a provides the chemical structure of the ferroelectric phase (*β*‐phase) PVDF‐TrFE, whose ferroelectricity originates from the electronegativity difference between the hydrogen and fluorine atoms that leads to dipole moment in a perpendicular direction to the main chain.^[^ ^13^ ^]^ Generally, the formation of *β*‐phase PVDF is ensured when the molar ratio of TrFE is above 11 mol%.^[^ ^14^ ^]^ In this work, we used the molar ratio of 70% (PVDF): 30% (TrFE), which results in spontaneous ferroelectricity in the film. Actually, a low annealing temperature of only 90 °C is required for the PVDF‐TrFE in our study, which is important to the fabrication of perovskite FETs as shown below.

To confirm the ferroelectricity of PVDF‐TrFE in nBA, we conducted X‐ray diffraction (XRD) studies on PVDF‐TrFE films. The obvious Bragg peak at 2*θ* of 19.9° corresponds to the (200) reflection, which is an indication of the *β* phase (Figure 1b).^[^ ^15^ ^]^ The film surface morphology was characterized by atomic force microscopy (AFM), which shows the grain size of PVDF‐TrFE up to 1 µm, indicating the good crystallinity of the film (Figure 1c). The thickness of the PVDF‐TrFE on silicon wafer and indium tin oxide (ITO) glass processed under the same conditions was determined to be 540 and 800 nm, respectively (see Figures S1 and S2, Supporting Information).

Furthermore, piezoresponse force microscopy (PFM) was employed to characterize the films by measuring the dynamic electromechanical response (see more details in Figure S3, Supporting Information).^[^ ^16^ ^]^ As shown in Figure 1d, the 180° phase contrast reversal was observed, which is attributed to the switching of the electric dipoles. Meanwhile, the variation of the amplitude as a function of voltage exhibits the butterfly shape, which is typical for ferroelectric materials as reported in previous studies.^[^ ^17^ ^]^ Notably, the switching voltages at the positive and negative regimes are asymmetric due to the usage of different electrodes during the PFM characterizations, which was commonly observed in previous works.^[^ ^18^ ^]^ In addition, we characterized the current–voltage (*I*–*V*) characteristics of the PVDF‐TrFE films by employing the sandwich structure: ITO/PVDF‐TrFE/Au (shown in the inset of Figure 1e). Two peaks appear at ±50 MV m^−1^ due to the 180° rotation of the electric dipoles induced by an applied electric field. All these results suggest the PVDF‐TrFE films processed from nBA are ferroelectric in nature.

Finally, we evaluated the capacitance‐frequency characteristics of PVDF‐TrFE films, which yield a capacitance (*C*_i_) value of about 26 nF cm^−2^ at 1 kHz and accordingly a dielectric constant of 10.5, in good agreement with literature reports (Figure 1f).^[^ ^19^ ^]^

### 2.2 Fabrication and Characterization of Semi‐Transparent Thin‐Film MAPbBr_3_ FETs

**Figure 2:**
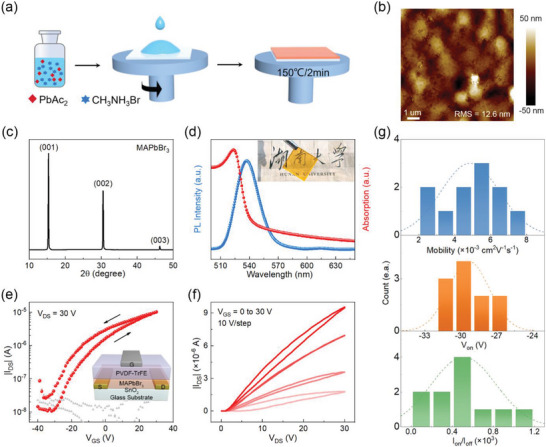
Electrical characterization of thin‐film MAPbBr_3_ FETs. a) The preparation process of MAPbBr_3_ film. b) AFM image of MAPbBr_3_ film. c) XRD patterns, d) UV–vis absorbance and steady‐state PL of MAPbBr_3_ film. The inset shows the semi‐transparent MAPbBr_3_ film. e) Transfer and f) output characteristics for MAPbBr_3_ FETs with ferroelectric dielectric PVDF‐TrFE. The inset shows the device structure. g) Histograms of saturation mobility, turn‐on voltage (*V*_on_), and on/off ratio (*I*_on_ */I*_off_) for eleven MAPbBr_3_ TGBC devices.

With the successful deposition of ferroelectric PVDF‐TrFE films by nBA solvent, we intend to utilize them for the construction of perovskite FETs. First, we show the fabrication and characterization of semi‐transparent FETs made of MAPbBr_3_ films. Here, we used lead acetate trihydrate (Pb(Ac)_2_) as the precursor of MAPbBr_3_ for film preparation to avoid the limited solubility of the usual perovskite precursor PbBr_2_ in classical solvents such as N,N‐dimethylformamide (DMF) and the rapid crystallization of perovskite films.^[^ ^20^ ^]^ The films were deposited by spin‐coating method, which is scalable for large‐area electronics (**Figure** **2**a). The morphology of MAPbBr_3_ films annealed at 150 °C for 2 min was characterized by AFM, with the image presented in Figure 2b. The film exhibits a typical polycrystalline structure, with root mean square (RMS) roughness of 12.6 nm. The film thickness is about 220 nm according to the AFM results (Figure S4, Supporting Information). XRD patterns show diffraction peaks at 15.2°, 30.6°, and 46.2°, which correspond to the (001), (002), and (003) planes, respectively (Figure 2c).^[^ ^21^ ^]^ The full‐width at half‐maximum (FWHM) of (001) peak is found to be 0.16°, indicating the high crystallinity of the MAPbBr_3_ film, and is consistent with the results reported in previous studies.^[^ ^21^, ^22^ ^]^ In addition, the UV–vis absorption spectrum of MAPbBr_3_ film shows a clear band edge at about 525 nm, and a narrow emission peak located at ≈540 nm is seen in steady‐state photoluminescence (PL) spectrum (Figure 2d), both of which point to a bandgap of ≈2.3 eV and thus a semi‐transparent film as shown in the inset of Figure 2d.

For the construction of TGBC FETs based on the MAPbBr_3_ films, glass substrates with defined Cr/Au electrodes by photolithography were used for the devices. Notably, prior to the deposition of MAPbBr_3_ films, a thin SnO_2_ interlayer (≈2 nm, Figure S5, Supporting Information) was deposited by spin‐coating process. This interlayer is aimed for improving the charge injection at the source/drain electrode interface because we see there is a significant barrier between the Fermi level of Au electrode (5.2 eV) and the conduction band (CB) of MAPbBr_3_ (≈3.38 eV).^[^ ^6^ ^]^ The PVDF‐TrFE dielectric was subsequently deposited on top of MAPbBr_3_ by spin‐coating process and annealed at 90 °C for 60 min, after which the Al gate electrode was evaporated through a shadow mask to complete the device.

Remarkably, the transfer curves of MAPbBr_3_ FETs exhibit well‐defined n‐type characteristics at RT when a continuous gate voltage was applied (Figure 2e). The gate leakage current is several orders of magnitude lower than the drain current, suggesting the reliable operation of the device. The output characteristics of the device are presented in Figure 2f, from which we see the lack of current saturation that is expected for ideal FETs. The reasons behind this phenomenon are not fully clear yet and deserve further studies. The statistical data of mobility, threshold voltage and on/off ratio for eleven devices is shown in Figure 2g. The champion device shows an on/off ratio of 10^3^ and mobility (*µ*) of 0.008 cm^2^ V^−1^ s^−1^.

It might be argued that the mobility for the devices is relatively low. However, we note there have been rare reports on MAPbBr_3_ FETs made from thin‐films, and the performance of our device is among the best. For instance, Wang et al. once fabricated thin‐film MAPbBr_3_ FETs yet the device mobilities are only on the order of 10^−3^ cm^2^ V^−1^ s^−1^ even at a low temperature of 100 K, and the hysteresis effects of those devices are very pronounced. It is even worse that no gate modulation was observed at temperatures above 240 K in those devices. They concluded that it is much more challenging to fabricate MAPbBr_3_ FETs compared to MAPbI_3_ FETs because MAPbBr_3_ is more prone to ion migration than MAPbI_3_ under similar process conditions.^[^ ^7b^ ^]^ Thus, it is arguable that we have for the first time obtained thin‐film MAPbBr_3_ FETs that can operate at room temperature with decent performance.

One notable thing is the anticlockwise hysteresis in the device characteristics (see Figure 2e), which is supposed to be caused by the ferroelectric property of PVDF‐TrFE.^[^ ^7^, ^8^ ^]^ Specifically, as the amplitude of the gate voltage increases, the electric dipoles in PVDF‐TrFE switch gradually, resulting in an increase in the remnant polarization, which reinforces the electric field in the channel to some extent, and thus the current becomes larger when the gate voltage is scanned backward.^[^ ^11^ ^]^

### 2.3 Fabrication and Characterization of Transparent Thin‐Film MAPbCl_3_ FETs

Having obtained semi‐transparent FETs made of MAPbBr_3_, we are encouraged to further pursue fully transparent FETs by using MAPbCl_3_ as the semiconductor layer, which is more challenging since the bandgap of MAPbCl_3_ is even larger than that of MAPbBr_3_. To the best of our knowledge, there have been no reports of FETs made of MAPbCl_3_ thin‐films, implying the huge challenges in fabrication of such devices.

**Figure 3:**
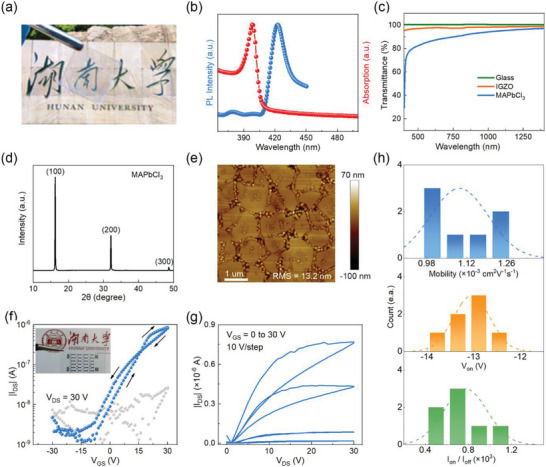
Characterization of thin‐film MAPbCl_3_ FETs. a) The photograph of MAPbCl_3_ film showing its transparent feature. b) UV–vis absorbance and steady‐state PL of transparent MAPbCl_3_ film. c) The transmittance spectra of the glass, IGZO and MAPbCl_3_ films. d) XRD patterns and e) AFM image of MAPbCl_3_ film. f) Transfer and g) output characteristics for MAPbCl_3_ FETs with ferroelectric dielectric PVDF‐TrFE. The inset shows the transparent feature of the devices. h) Histograms of saturation mobility, turn‐on voltage, and on/off ratio for seven MAPbCl_3_ TGBC devices.

We first tackled the problem of obtaining high‐quality MAPbCl_3_ films. Unlike MAPbBr_3_ films which can be processed well by a single solvent DMF, the MAPbCl_3_ films processed by DMF alone show rough surface morphologies (see Figure S6, Supporting Information). Thus, for the deposition of MAPbCl_3_ films, we added a portion of dimethyl sulfoxide anhydrous (DMSO) to the previously mentioned perovskite solvent DMF to retard the rapid reaction between PbAc_2_ and MACl during the spin‐coating process,^[^ ^23^ ^]^ which is helpful to improve the morphology of the films. **Figure** **3**a presents the photograph of the MAPbCl_3_ film deposited on glass substrate, which has a thickness of about 90 nm (Figure S7, Supporting Information) and looks highly transparent (Figure S8, Supporting Information). The UV–vis absorption spectrum of MAPbCl_3_ films shows a clear band edge at about ≈400 nm, and a narrow emission peak of steady‐state photoluminescence (PL) spectrum located at ≈423 nm, which yields a bandgap of 3.0 eV that is responsible for the transparent property of MAPbCl_3_ (Figure 3b). To quantitatively understand the transparency of the MAPbCl_3_ film, we measured the transmittance of MAPbCl_3_, which shows the transparency of 80–90% in the wavelength range of 400 to 1400 nm. This transparency value is only slightly lower than that of the typical transparent oxide semiconductor IGZO, which has a transparency of more than 90% between 400 and 1400 nm (Figure 3c). These results suggest MAPbCl_3_ is promising for applications in next‐generation “invisible” displays, and smart windows.^[^ ^24^ ^]^

To determine the structure of MAPbCl_3_ films prepared by the above method, XRD characterization was performed and the pattern of MAPbCl_3_ clear shows three main diffraction peaks at 16.3°, 32.2°, 48.7° corresponding to (100), (200), and (300) planes, respectively (Figure 3d).^[^ ^12^, ^24^ ^]^ The film morphologies were characterized by AFM, with results shown in Figure 3e. It is seen the film exhibits typical polycrystalline structure with uniform grains and disordered grain boundaries. Although the film as whole can form conductive path for charge carriers, some voids with ultrathin or no films are observed in the grain boundaries (see Figure S7, Supporting Information). These boundaries are likely to seriously disturb charge transport although their effects remain to be further investigated.

By adopting the similar fabrication procedures used for MAPbBr_3_ FETs, i.e., the usage of the ferroelectric PVDF‐TrFE as the dielectric and SnO_2_ as the interlayer between electrodes and perovskites, transparent TGBC MAPbCl_3_ FETs were obtained, as shown in the inset of Figure 3f. The transfer and output curves show clear n‐type transistor characteristic (Figure 3f,g), with electron mobility of 1.25 × 10^−3^ cm^2^ V^−1^ s^−1^ and on/off ratio of 10^3^. The statistical distribution of electron mobility, turn‐on voltage and on/off ratio for MAPbCl_3_ devices is shown in Figure 3h. Although the performance of these devices is relatively low compared to that of IGZO FETs, these devices provide alternatives and new technique routes for the development of transparent semiconductor devices, and they are particularly attractive in terms of the low‐temperature (≤150 °C) solution processability. Moreover, the morphology of the MAPbCl_3_ films suggests that higher performance can be expected once the film morphology/structure is further improved.

Interestingly, a complex hysteresis behavior was observed in the transfer characteristic of MAPbCl_3_ FETs; the hysteresis is clockwise at voltage higher than about 18 V but anticlockwise below that voltage (see Figure 3f). This complex hysteresis behavior is suspected to be resulted from the combined interaction of ferroelectric effect and ion‐migration/trapping effect. In specific, the ferroelectric dielectric PVDF‐TrFE is supposed to cause anticlockwise hysteresis as seen in MAPbBr_3_ FETs, while the ion‐migration/trapping effect generally results in clockwise hysteresis in n‐type perovskite FETs.^[^ ^8b^ ^]^ We identify the clockwise hysteresis in MAPbCl_3_ FETs is mainly attributed to ion‐migration effect by measuring the transfer curves at difference scanning rates (see Figure S11, Supporting Information). Thus, the hysteresis is anticlockwise due to ferroelectric effect at low gate bias but clockwise when ion migration effect is dominant at high gate voltages.

### 2.4 Understanding the Operation Principles of Ferroelectric MAPbX_3_ (X = Br, Cl) FETs

The demonstration of MAPbBr_3_ and MAPbCl_3_ thin‐film transistors that function well at RT apparently represents significant progress of MHP FETs. Essentially, it is the device fabrication strategy we proposed that leads to such progress. In this section, we intend to understand the effect of the strategy on device operation and performance.

**Figure 4:**
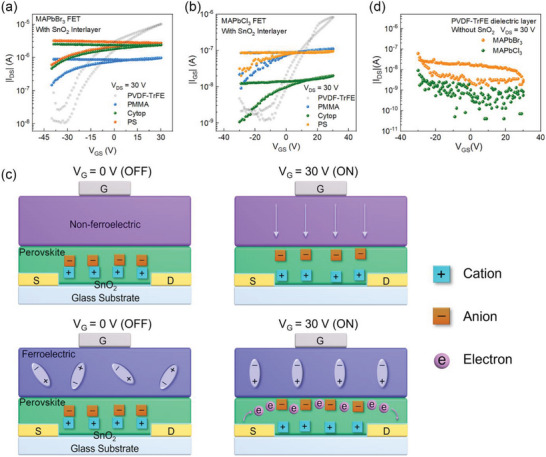
Investigation on the operation principles of ferroelectric MAPbBr_3_ and MAPbCl_3_ FETs. The transfer curves of a) MAPbBr_3_ FETs and b) MAPbCl_3_ FETs with non‐ferroelectric dielectrics. c) The schematic diagram illustrating the difference between the perovskite FETs with ferroelectric dielectric and non‐ferroelectric dielectrics. d) The performance of MAPbX_3_ (X = Br and Cl) FETs with PVDF‐TrFE dielectric layer but no SnO_2_ interlayer.

First, we show the ferroelectric PVDF‐TrFE plays a fundamental role in these devices, which can be apparently seen in the control devices with non‐ferroelectric dielectrics. As shown in **Figure** **4**a, the MAPbBr_3_ devices with polymer dielectrics polystyrene (PS), poly(methyl methacrylate) (PMMA) and Cytop show weak modulation of gate voltage over current, which is commonly seen in MHP FETs due to the screening of gate electric field by ion migration effect.^[^ ^7b^ ^]^ Similarly, MAPbCl_3_ devices fabricated with these non‐ferroelectric dielectrics show no transistor behaviors at RT (Figure 4b). These results suggest the indispensability of the ferroelectric PVDF‐TrFE for the operation of the devices. In addition, it should be mentioned that PVDF‐TrFE is not the only choice for MAPbBr_3_/MAPbCl_3_ FETs, other ferroelectric polymers such as poly(vinylidene fluoride‐co‐hexafluoropropylene) (PVDF‐HFP) can also result in operation of MAPbBr_3_/MAPbCl_3_ FETs at RT (see Figure S13, Supporting Information).

Previously, it was reported methylammonium lead halide (MAPbI_3_) exhibits a surface ion density of 5 µC cm^−2^.^[^ ^25^ ^]^ Such a large charge density would demand the application of gate voltages greater than 1000 V in order to induce an accumulation channel for a typical polymer gate dielectric with the capacitance of about 5 nF cm^−2^, which is impractical since the high gate voltage would cause a dielectric breakdown. Nevertheless, ferroelectric dielectric materials like PVDF‐TrFE are capable of inducing surface charge density as large as 7–8 µC cm^−2^ due to the local strong electric field of dipoles.^[^ ^11b^ ^]^ Therefore, an accumulation charge conduction channel can be formed upon the usage of a ferroelectric dielectric, even though the gate‐electric‐field screening effect caused by ion migration still exists in the perovskites. We believe this is the main reason accounting for the operation of the ferroelectric perovskite FETs at RT in this study, and moreover, this strategy should be generally applicable to other perovskite FETs for helping overcome the gate‐electric‐field screening problem.

Figure 4c schematically shows the difference between the FETs with non‐ferroelectric and ferroelectric dielectrics. For non‐ferroelectric perovskite FETs, when a positive gate voltage is applied to the gate, the halide ions (Br^−^ or Cl^−^), which can migrate upon the application of electric field, will drift toward the semiconductor/dielectric interface, screening the gate voltage and thus no electron transporting channel can be formed. In contrast, for ferroelectric perovskite FETs, the dipoles are polarized down when a positive gate bias voltage is applied. In this case, the halide ions will still move to the semiconductor/dielectric interface. However, there would still be electrons accumulated at the interface besides ions because the polarized dipoles can induce a high density of surface charge.

In addition, we inspected the effect of the SnO_2_ interlayer on the performance of the devices. One undeniable fact is that the absence of SnO_2_ will disable the device operation at RT, as illustrated in Figure 4d. Thus, the SnO_2_ interlayer is necessary for the fabrication of MAPbBr_3_ and MAPbCl_3_ FETs. Also, we assured that the SnO_2_ interlayer itself cannot result in transistor characteristics, as shown in Figure S16 (Supporting Information). We further investigated the possible influences of SnO_2_ on the crystallinity and morphology of the perovskite film deposited atop. However, it seems such influences are negligible even if there are any (see Figures S18–S20, Supporting Information). These results suggest that the SnO_2_ layer very likely acts as an interlayer for facilitating electron injection since SnO_2_ is well‐known to be an electron transporting layer in perovskite solar cells.^[^ ^26^ ^]^ Alternatively, the SnO_2_ layer may prevent the electrochemical reactions at the interface between the perovskite and Au source/drain contacts, which is believed to be detrimental to device performance.^[^ ^7b^ ^]^

## 3 Conclusion

To summarize, we have proposed the strategy of employing ferroelectric polymer dielectric PVDF‐TrFE and SnO_2_ interlayer for fabrication of MHP FETs. The usage of the ferroelectric dielectric PVDF‐TrFE addresses the well‐known challenge of the gate‐electric‐field screening effect faced by perovskite FETs, and the SnO_2_ interlayer enables the electron injection from metal electrodes into wide‐bandgap semiconductor such as MAPbCl_3_. Consequently, we have for the first time successfully obtained semi‐transparent MAPbBr_3_ FETs and fully transparent MAPbCl_3_ FETs that can operate well at RT. It is of importance these devices were fabricated with low‐temperature (≤150 °C) solution‐process, which is appropriate for device fabrication on almost any plastic substrates. These results may open up new opportunities to the fabrication and development of transparent electronics.

## 4 Experimental Section

### Materials

Methylammonium bromide (MABr, ≥99.5%), methylammonium chloride (MACl, ≥99.5%) were purchased from Xi'an Polymer Light Technology Corp. Poly(vinylidenefluoridetrifluoroethylene) (P(VDF‐TrFE), 70:30 mol%) was purchased from Piezotech. Lead acetate trihydrate (PbAc_2_, 99.998), polystyrene (PS, MW ≈ 280 000), polymethylmethacrylate (PMMA, MW ≈ 350 000), chlorobenzene (CB, 99.8%), n‐butyl Acetate (nBA, 99.5%), and *N*,*N*‐dimethylformamide (DMF, anhydrous, 99.8%) were purchased from Sigma‐Aldrich. Dimethyl sulfoxide anhydrous (DMSO, anhydrous, 99.8%) was purchased from Alfa Aesar. Cytop solution was prepared by adding Cytop solvent into Cytop with a volume ratio of 1:3. SnO_2_ solution was obtained by mixing SnO_2_ colloidal and deionized water in a volume ratio of 1:6. All materials were used as received without any additional purification.

### Preparation of MAPbX_3_ (X = Br and Cl) Films

0.75 m MAPbBr_3_ precursor solution was fabricated from a precursor mixed solution of PbAc_2_/MABr (1:3) in DMF. For MAPbCl_3_ precursor solution, 0.75 m solution of PbAc_2_/MACl (1:3) in DMSO was first prepared, and then mixed with DMF at a volume ratio of 4:1. MAPbCl_3_ precursor solutions were heated at 45 °C for 10 h in an Ar‐filled glovebox and filtered through 0.45 µm PTFE filters before usage. The MAPbX_3_ (X = Br and Cl) films were prepared by spin‐coating the precursor solution on the substrates at 4000 rpm for 30 s. All perovskite films were annealed at 150 °C for 2 min in the Ar‐filled glovebox. In this annealing process, the evolution of these materials can be described by the equation:

(1) $$PbAc_{2}+3CH_{3}NH_{3}X\to CH_{3}NH_{3}PbX_{3}+2CH_{3}NH_{3}Ac(X=Br,Cl$$

The by‐product (CH_3_NH_3_Ac) of the above transition will evaporate during the annealing process.

### Fabrication of MAPbX_3_ FETs

Top‐gate, bottom‐contact (TGBC) FETs were fabricated on Cr/Au (5 nm: 30 nm) S/D electrodes that were evaporated and patterned photolithographically on precleaned, UV/ozone treated glass substrates. The SnO_2_ layer was deposited by spin‐coating at 3000 rpm for 30 s and then annealed at 150 °C for 30 min to completely remove the aqueous solvent. This was followed by the deposition of the perovskite film as described above in an Ar‐filled glovebox. The dielectric layers of PMMA (70 mg mL^−1^, dissolved in nBA), PS (100 mg mL^−1^, dissolved in nBA), Cytop and P(VDF‐TrFE) (70 mg mL^−1^, dissolved in nBA) were then spin‐coated over the perovskite film. PMMA, PS and Cytop were deposited at 1500 rpm for 20 s and then annealed at 90 °C for 20 min. PVDF‐TrFE was cast uniformly onto the perovskite samples and spin‐coated with two programmed steps, the first at 500 rpm for 3 s, and the second at 4000 rpm for 20 s. Post‐annealing at 90 °C for 1 h was used to enhance film quality. For the gate electrode, 50 nm thick Al electrode was thermally evaporated (1.5 Å s^−1^) through a shadow mask.

### Characterization of Films

The film morphology was measured by SEM (TESCAN MIRA3) and atomic force microscopy (AFM) (Bioscope system). The thickness of films was measured by profilometer (XP‐200, Ambios). The X‐ray diffraction (XRD) patterns were recorded by D/max 2550 (Rigaku) under Cu K*α* (*λ* = 1.5406 Å) irradiation in the ambient atmosphere. PL spectra were recorded by a Thermo Scientific Lumina. The ultraviolet–visible–near‐infrared (UV–vis–NIR) absorption spectra of perovskite films were characterized with UV‐3600 PLUS (Shimadzu). The P‐E hysteresis loops were measured by a ferroelectric characterization system (TF‐Analyzer 1000, aixACCT, Germany), and the frequency of the voltage pulse was 1000 Hz. The local piezoresponse force microscopy (PFM) measurements were performed with an Asylum Research Cypher scanning probe microscope (Asylum Research, Oxford Instruments) using the Nanosensors PPP‐EFM chromium/platinum‐iridium (Cr/Pt‐Ir)‐coated silicon cantilevers, whose radius were ≈25 nm. The capacitance of P(VDF‐TrFE) was carried out using LCR digital electric bridge (TH2838).

### Measurement of MAPbX_3_ FETs

Electrical properties of TGBC FETs were performed with a Keysight B2912A Precision Sources through a probe station in an Ar glovebox. All measurements of perovskite FETs were carried out under dark conditions. The channel length and width of FET were 40 and 2000 µm, respectively. It should be noted that all the measurement were performed in continuous DC mode rather than pulse mode.

### Statistical Analysis

The performance data of the perovskite FETs for n devices (*n* = 7 or 11) were summarized and presented as a distribution graph without pre‐processing. The mean or standard deviation values were not calculated. The data processing was performed by Origin software.

## Conflict of Interest

The authors declare no conflict of interest.

## Supporting information

Supporting Information

## Data Availability

The data that support the findings of this study are available from the corresponding author upon reasonable request.
