# Supplementary material for: Ferroelectric Wide‐Bandgap Metal Halide Perovskite Field‐Effect Transistors: Toward Transparent Electronics
Source: Adv Sci (Weinh). 2023 Jan 26;10(10):2300133. doi: 10.1002/advs.202300133 (PMC10074105; doi:10.1002/advs.202300133)
Supplement: Supplementary file 1 — Supporting Information [file ADVS-10-2300133-s001.pdf]

ADVANCED SCIENCE
Open Access

Supporting Information

for *Adv. Sci.*, DOI 10.1002/advs.202300133

Ferroelectric Wide-Bandgap Metal Halide Perovskite Field-Effect Transistors: Toward Transparent Electronics

Jiangnan Xia, Xincan Qiu, Yu Liu, Ping-An Chen, Jing Guo, Huan Wei, Jiaqi Ding, Haihong Xie, Yawei Lv, Fuxiang Li, Wenwu Li, Lei Liao and Yuanyuan Hu**

Supplementary Information

Ferroelectric Wide-Bandgap Metal Halide Perovskite Field-Effect Transistors: Towards Transparent Electronics

Jiangnan Xia[1,2,3], Xincan Qiu[1], Yu Liu[1], Ping-An Chen[1], Jing Guo[1], Huan Wei[1], Jiaqi Ding[1], Haihong Xie[1], Yawei Lv[1], Fuxiang Li[1], Wenwu Li[4*], Lei Liao[3], Yuanyuan Hu[1,2,3*]

[1]Key Laboratory for Micro/Nano Optoelectronic Devices of Ministry of Education, School of Physics and Electronics, Hunan University, Changsha 410082, China
[2]Shenzhen Research Institute of Hunan University, Shenzhen 518063, China
[3]International Science and Technology Innovation Cooperation Base for Advanced Display Technologies of Hunan Province, College of Semiconductors (College of Integrated Circuits), Hunan University, Changsha 410082, China
[4]Shanghai Frontiers Science Research Base of Intelligent Optoelectronics and Perception, Institute of Optoelectronics, Department of Materials Science, Fudan University, Shanghai 200433, China

Corresponding authors: liwenwu@fdu.edu.cn; yhu@hnu.edu.cn

1. Characterizations on PVDF-TrFE films.

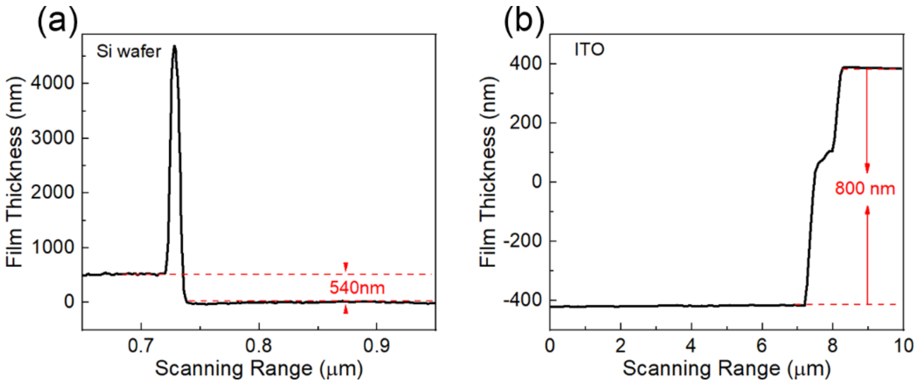

Figure S1. The thickness of P(VDF-TrFE) deposited on (a) silicon and (b) ITO/glass substrates measured by profilometer (XP-200, Ambios).

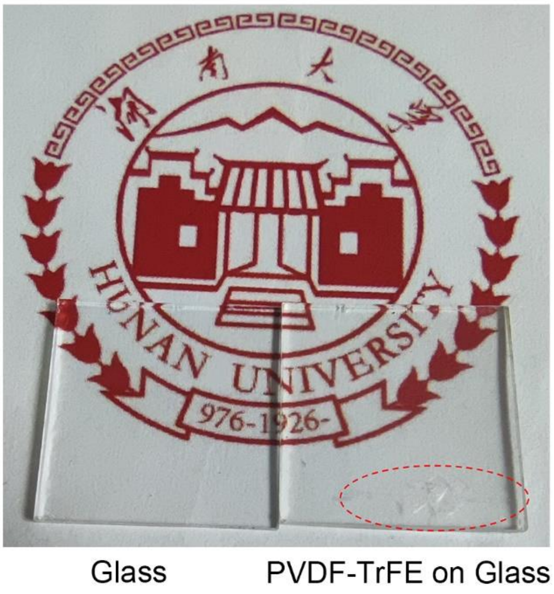

Figure S2. The photographs of the PVDF-TrFE film showing the transparency of it. The one on the left is a clean glass substrate and the one on the right is a glass substrate coated with PVDF-TrFE film. The red dotted line marks PVDF-TrFE thin film cut/peeled by a tweezer.

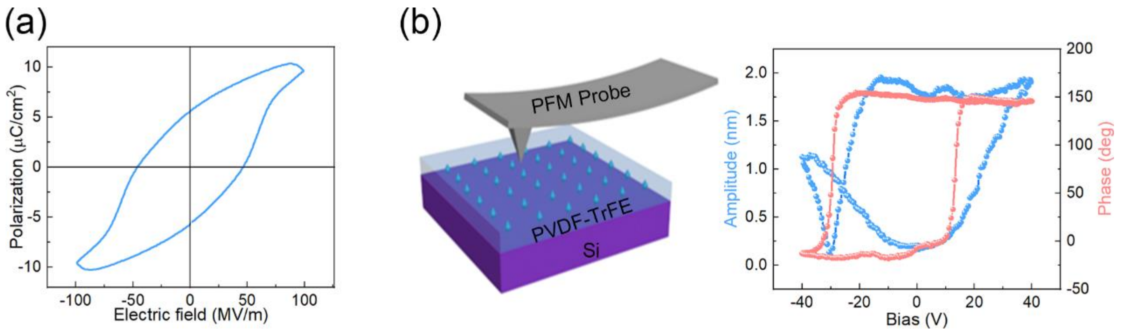

Figure S3. (a) The P-E hysteresis loop for ITO/PVDF-TrFE/Au diode. (b) Hysteresis behavior of PVDF-TrFE observed in the PFM phase and amplitude signals.

2. Characterizations on $MAPbBr_3$ films and devices.

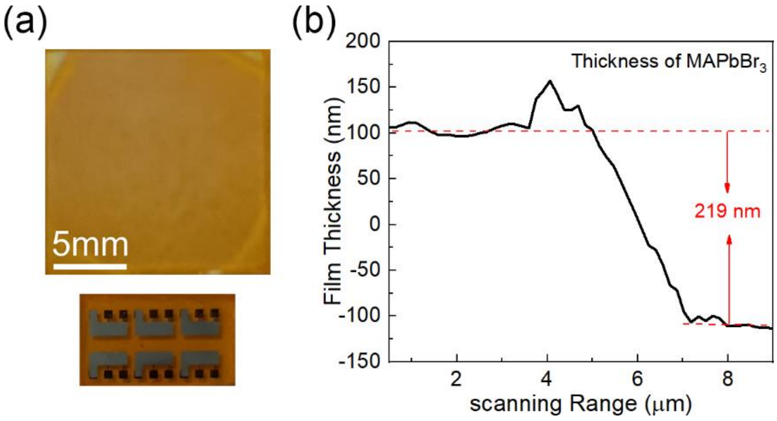

Figure S4. (a) The photograph of $MAPbBr_3$ film and TGBC $MAPbBr_3$ FET. (b) The thickness of the $MAPbBr_3$ film determined by AFM.

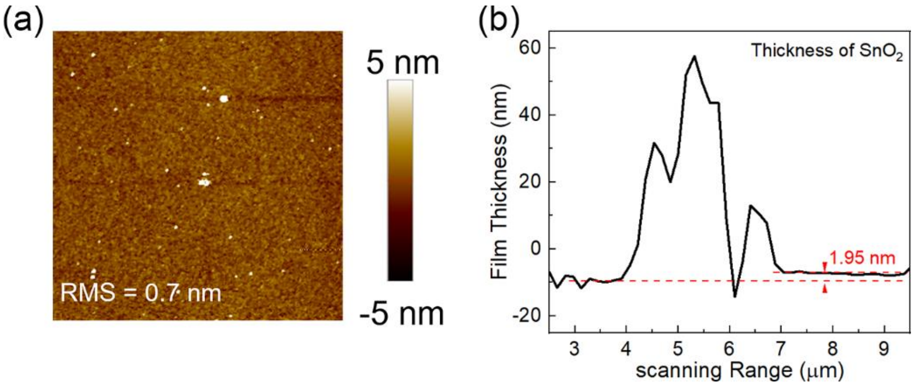

Figure S5. (a) The morphology and (b) thickness of SnO_2 film determined by AFM.

3. Characterizations on $MAPbCl_3$ films and devices.

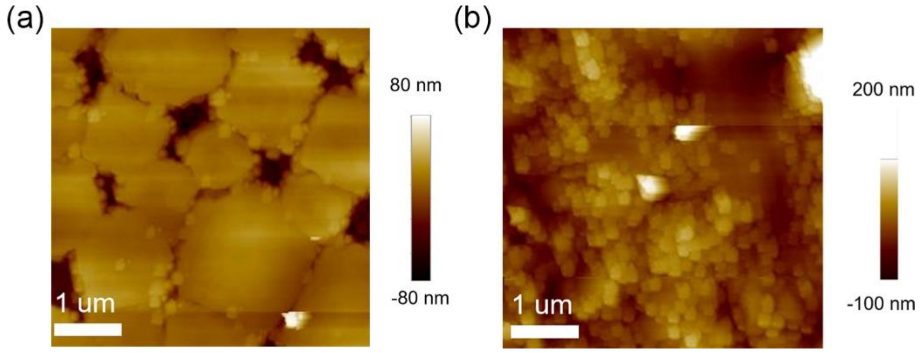

Figure S6. The morphology of the $MAPbCl_3$ films processed by solvent (a) DMSO and (b) DMF.

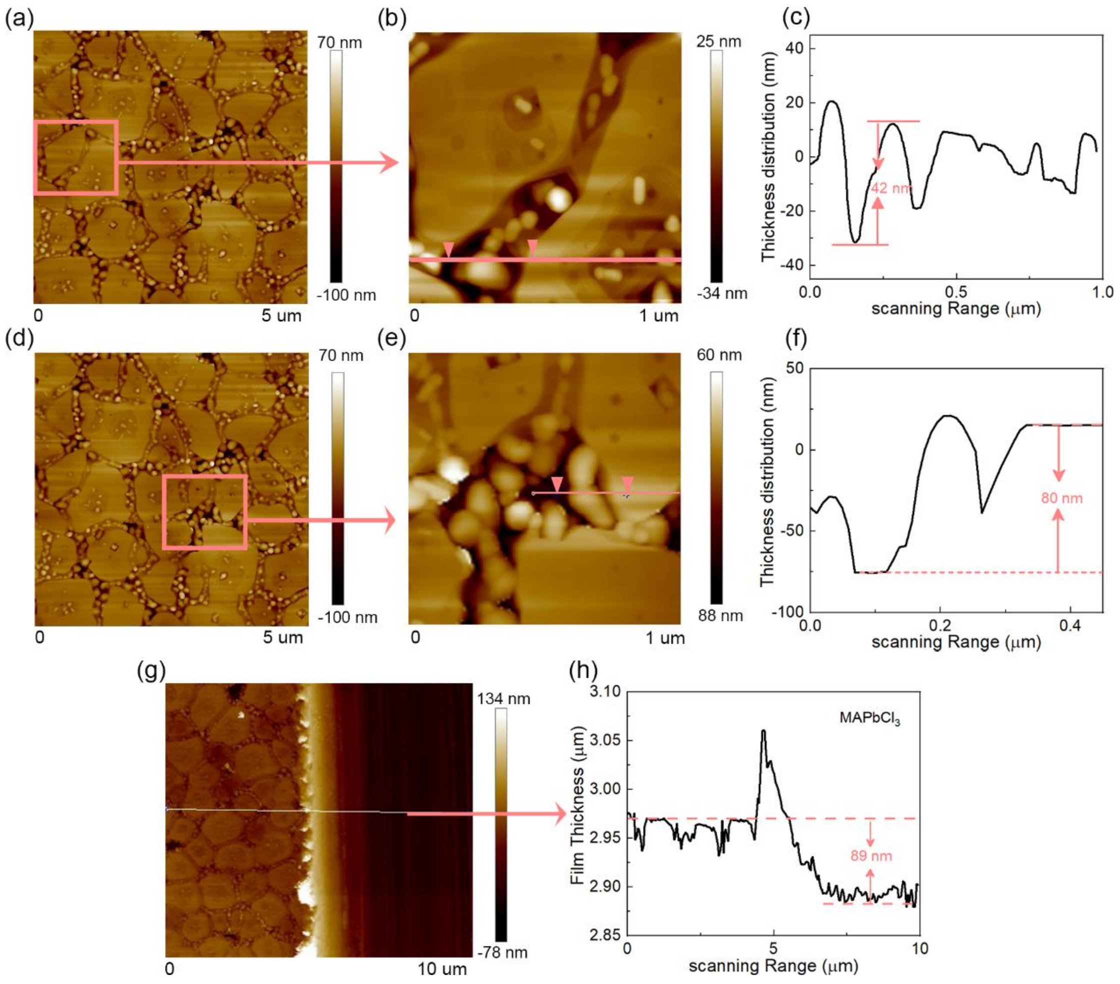

Figure S7. The high-resolution atomic force microscopy (AFM) of $MAPbCl_3$ films deposited on glass substrates. (a) The AFM image with size of 5 μm. The zoomed-in image (b) and line profile (c) of the parts marked in a. (d) The AFM image with size of 5 μm. The zoomed-in image (b) and line profile (c) of the parts marked in d. (e) The AFM image of the film edge and (h) the line profile showing the film thickness.

We zoomed in two types of grain boundaries: type I (the marked region in Figure S7a) and type II (the marked region in Figure S7d). As indicated by the line profile in Figure S7b, there is trough area with thickness of about 42 nm in the type I boundary. On the other hand, the film thickness is about 89 nm as shown in Figure S7g and h, which means that the type I boundary connect grains around. The line profile for the type II boundary is shown in Figure S7f, showing troughs with thickness of about 80 nm, which indicates they are voids with no films. The films are probably disconnected in such boundaries. Despite of the voids, we can still see that the film is connected as a whole, and charge carrier conduction path can be formed in such films, which rationalizes their usages as channel layer for FETs.

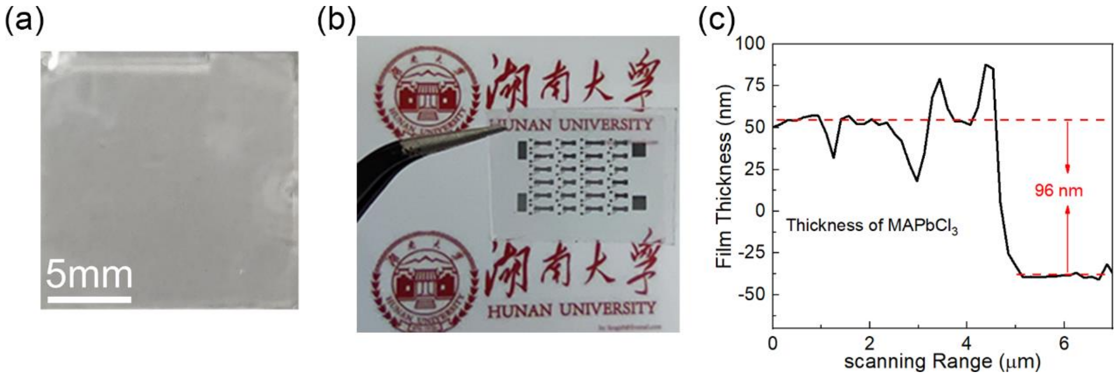

Figure S8. The photograph of (a) $MAPbCl_3$ film and (b) the top-gate, bottom-contact (TGBC) transparent $MAPbCl_3$ FET. (c) The thickness of $MAPbCl_3$ film determined by AFM.

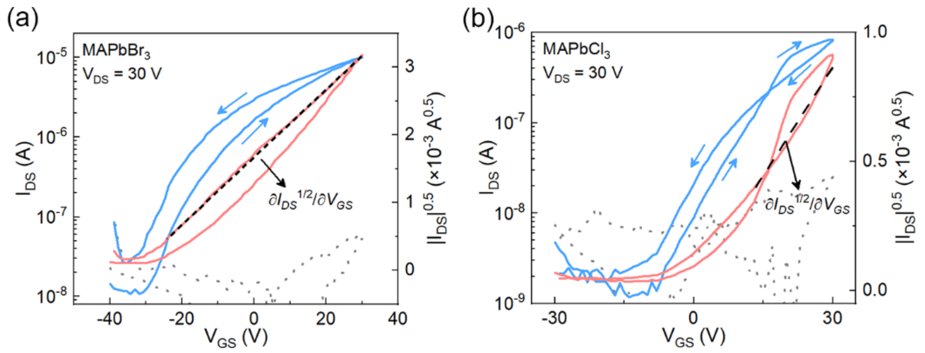

Figure S9. The graph showing mobility extraction for (a) $MAPbBr_3$ FETs and (b) $MAPbCl_3$ FETs.

The mobility reported in this study was extracted from the saturated region by the following equation:

$$\mu = \frac{2L}{C_i W}\left(\frac{\partial\sqrt{I_{DS}}}{\partial V_{GS}}\right)^2$$

Here, L, $C_{i,}$ and W are the length of the channel, the per unit area capacitance of the dielectric layer, and the width of the channel, respectively. To be conservative with the mobility values, we calculated the slope of $\left(\frac{\partial\sqrt{I_{DS}}}{\partial V_{GS}}\right)$ from the curves with lower slopes as shown in **Figure S9**.

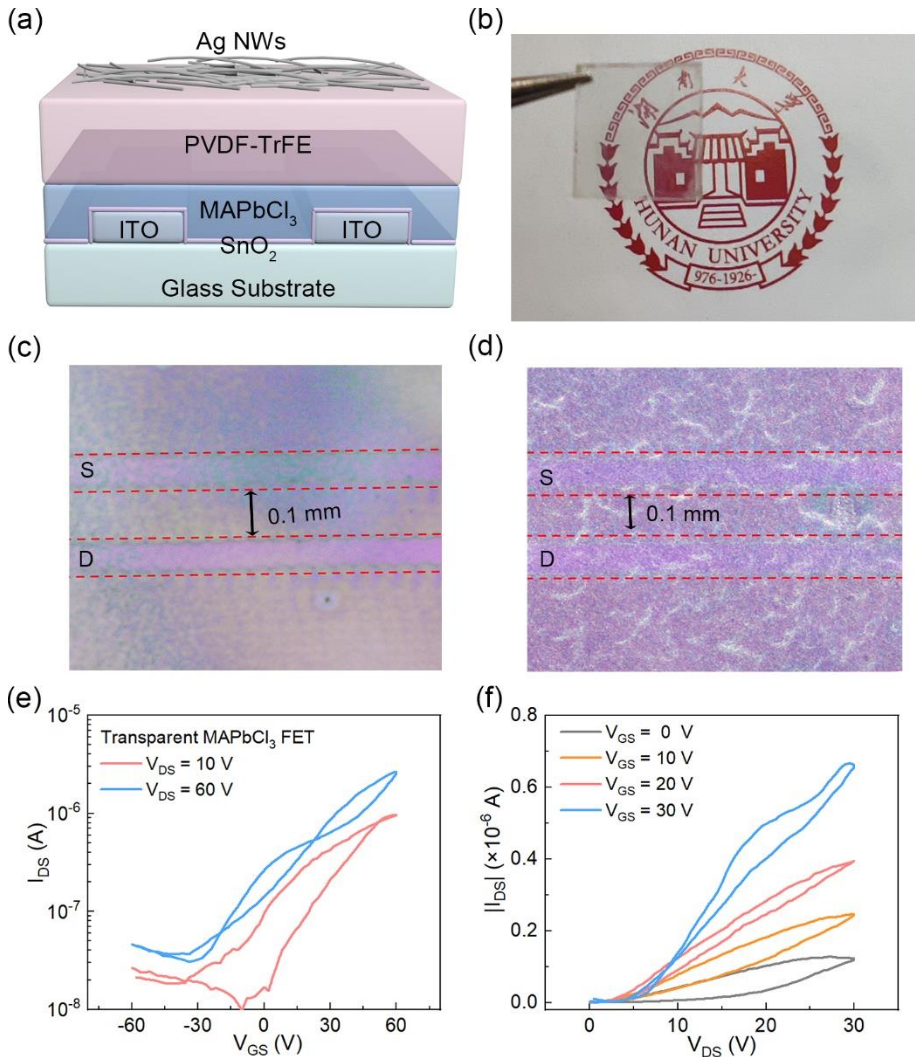

Figure S10. Fabrication and characterization of completely transparent TGBC $MAPbCl_3$ FETs with ITO as source/drain electrodes and Ag nanowires as gate electrodes. (a) Schematic diagram and (b) photograph of the transparent TGBC $MAPbCl_3$ device. (c) Bright field and (d) dark field optical microscope of the $MAPbCl_3$ FET. The width of the channel source/drain (ITO) is 0.1mm. The (e) transfer and (f) output curves of the transparent $MAPbCl_3$ FET. The ITO electrodes were purchased with patterns and directly used. The Ag nanowires (5 mg/mL in isopropanol, purchased from Suzhou ColdStones Technology Co) was spin-coated at 1500 rpm for 30 s and annealed at 70 ℃ for 1 min.

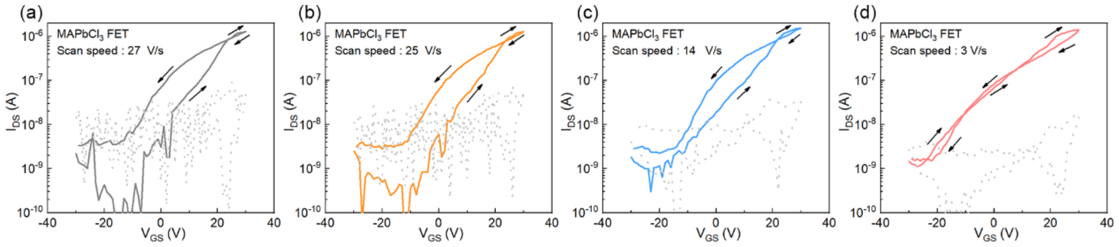

Figure S11. The transfer curves of $MAPbCl_3$ FET measured with different scanning rates, which indicates information about the origin of the hysteresis.

Basically, there are three effects that can cause hysteresis in our ferroelectric perovskite FETs: the ferroelectric effect, the ion-migration effect and the trapping effect. For n-type FETs, the ferroelectric effect is supposed to cause anticlockwise hysteresis, while the ion migration/trapping effect generally results in clockwise hysteresis. Thus, it is relatively easy to discriminate the ferroelectric effect from ion-migration/trapping effect. To differentiate ion-migration effect from trapping effect, we can measure the transfer curves at different voltage scanning rates. For ions which move slowly, they cannot response timely if the voltage scanning rate is fast. Thus, the faster the scanning rate, the lower the hysteresis. On the other hand, if charge carriers are trapped, they cannot de-trap timely if the voltage scanning rate is fast, and so hysteresis would appear if the scanning rate is fast but minimized if the scanning rate is low enough.

A clockwise hysteresis is observed at the high gate bias range, and this hysteresis becomes more apparent with the lowering of the scanning rate, suggesting such hysteresis is mainly caused by the ion-migration effect. It is reasonable to assume that the hysteresis effect caused by ferroelectric effect is not dependent on scanning rates because the polarization of dipoles induced by gate bias occurs fast. Thus, with the lowering of scanning rates, the hysteresis caused by ion-migration effect gets stronger, while the hysteresis caused by ferroelectric effect remains almost constant. The two effects may counterbalance each other and the device exhibits a reduced hysteresis, as seen in Figure S11d.

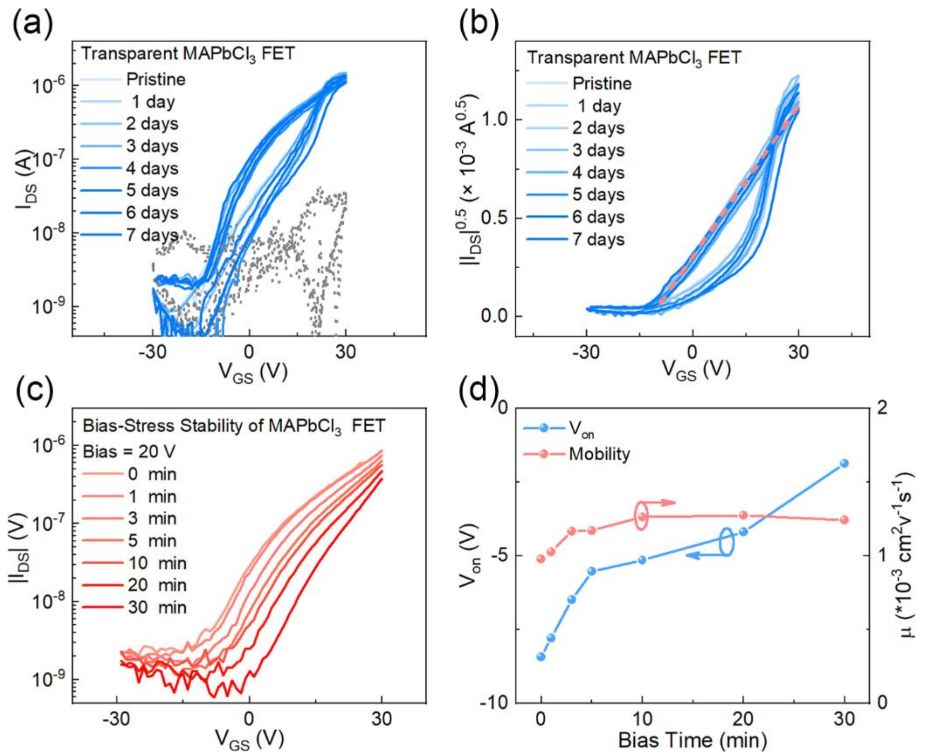

Figure S12. Evolution of the (a) transfer curve and (b) their corresponding square root I_{DS} versus V_G plots of $MAPbCl_3$ FETs within one week. (c) Transfer and (d) turn-on voltage, mobility of $MAPbCl_3$ FETs recorded at different times during bias-stress process (V_{GS} = 20 V and V_{DS} = 0 V).

4. Understanding the effect of ferroelectric dielectric.

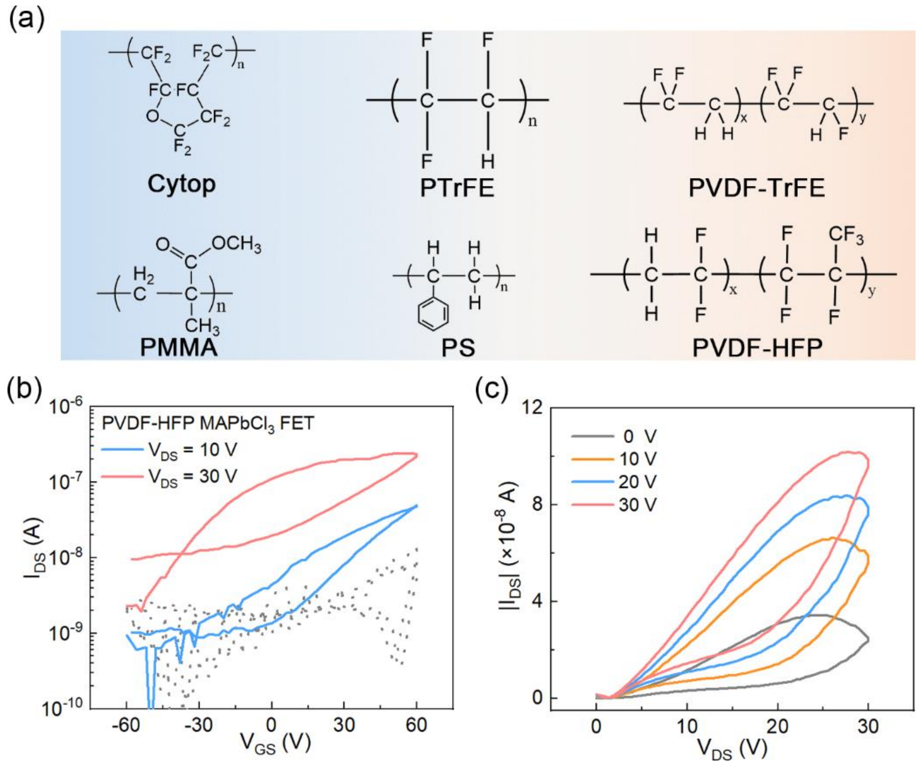

Figure S13. (a) Chemical structures of Cytop, PMMA, PTrFE, PS, PVDF-TrFE, and PVDF-HFP. The (b) transfer and (c) output curves of $MAPbCl_3$ FETs with PVDF-HFP dielectric layer.

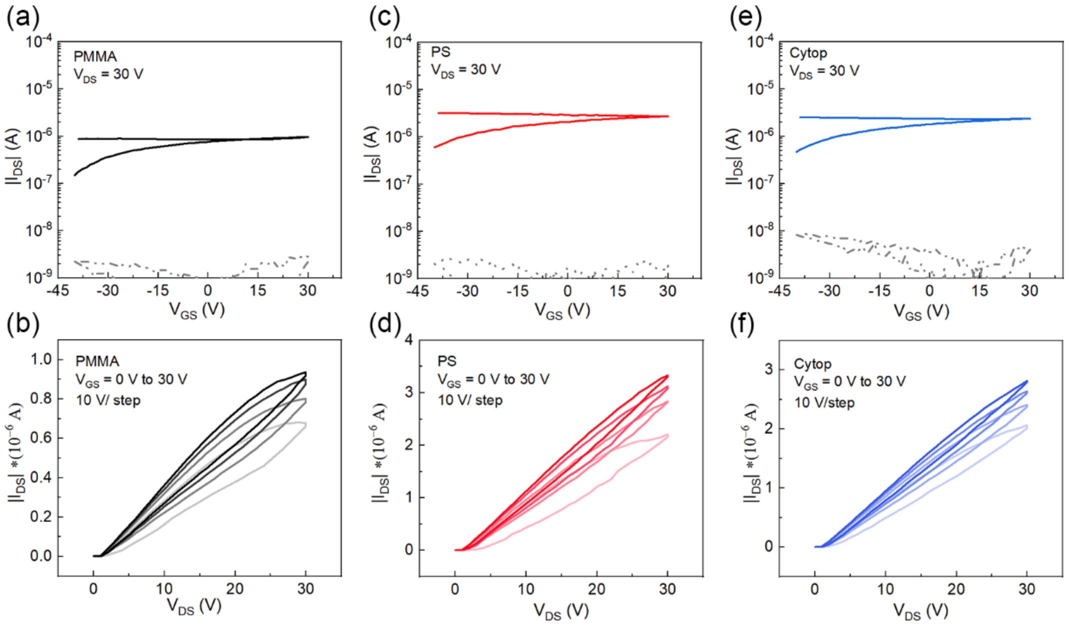

Figure S14. The transfer and output curves of the $MAPbBr_3$ FETs with different dielectric layer (with SnO_2 interlayer): (a) (b) PMMA, (c) (d) PS and (e)(f) Cytop.

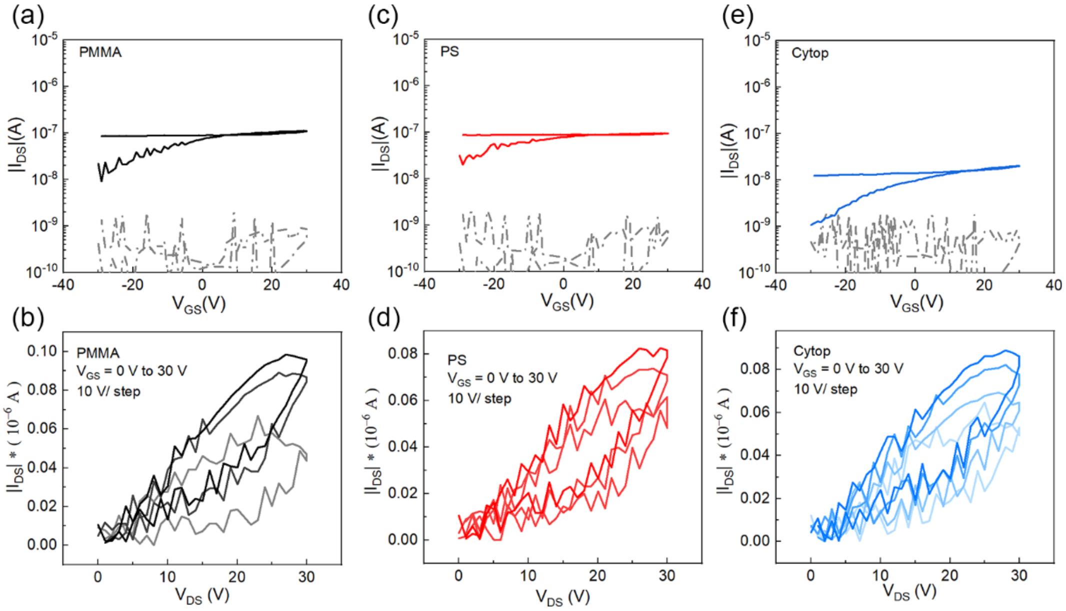

Figure S15. The transfer and output curves of the $MAPbCl_3$ FETs with different dielectric layer (with SnO_2 interlayer): (a) (b) PMMA, (c) (d) PS and (e)(f) Cytop.

5. Understanding the effect of SnO_2 interlayer.

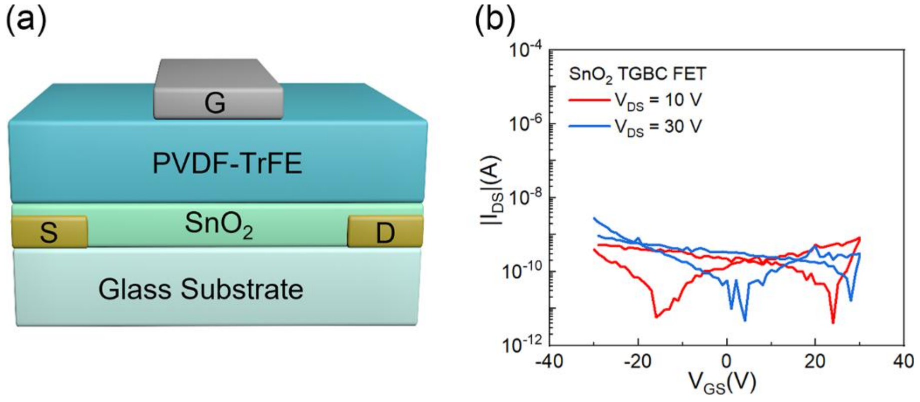

Figure S16. (a) Schematic diagram of the TGBC device with only SnO_2 layer. (b) The transfer curves of the SnO_2 FETs using PVDF-TrFE as the dielectric layer, indicating that the SnO_2 alone cannot result in current.

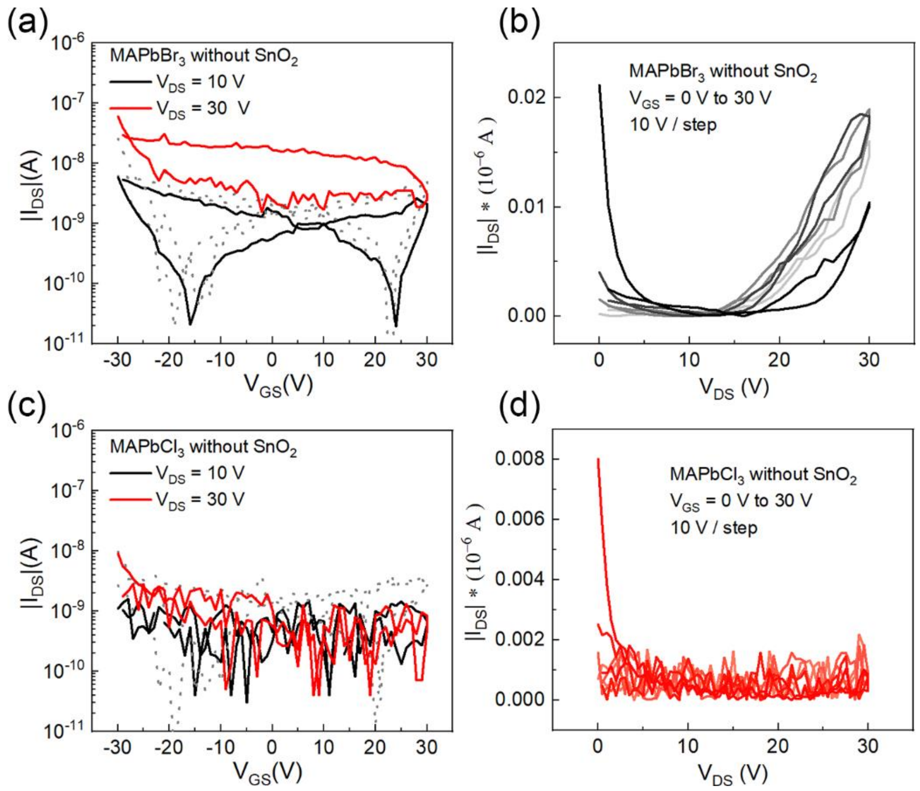

Figure S17. (a) The transfer and (b) output curves of $MAPbBr_3$ FETs without SnO_2 interlayer. (c) The transfer and (d) output curves of $MAPbCl_3$ FETs without SnO_2 interlayer. These results suggest that SnO_2 interlayer is required for the operation of these devices.

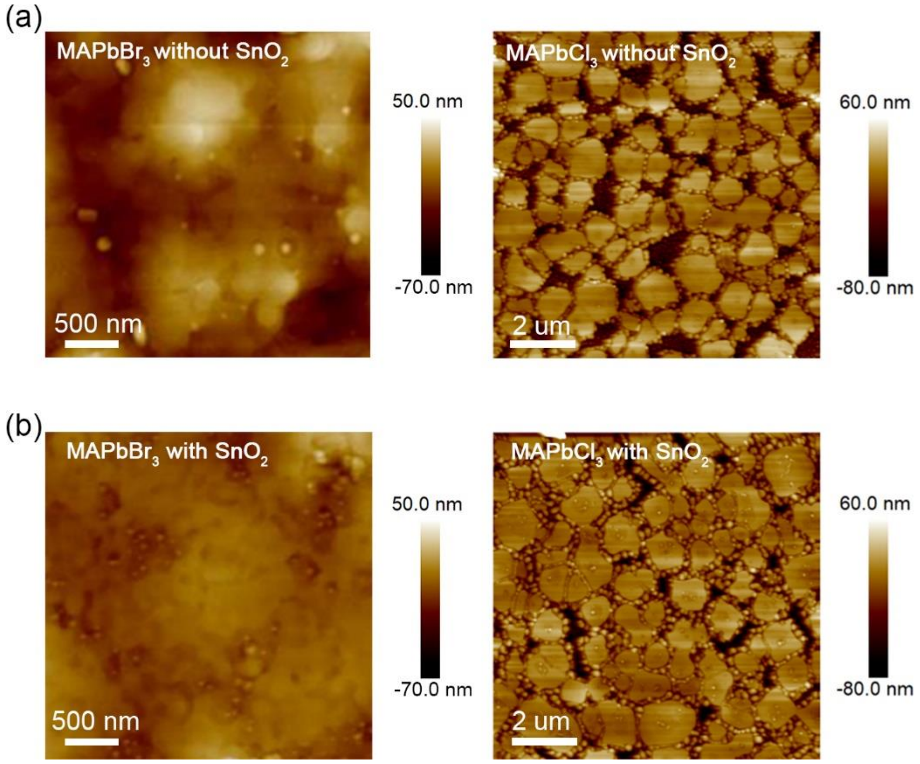

Figure S18. The AFM characterization results of $MAPbBr(Cl)_3$ films deposited on substrates (a) without SnO_2 and (b) with SnO_2.

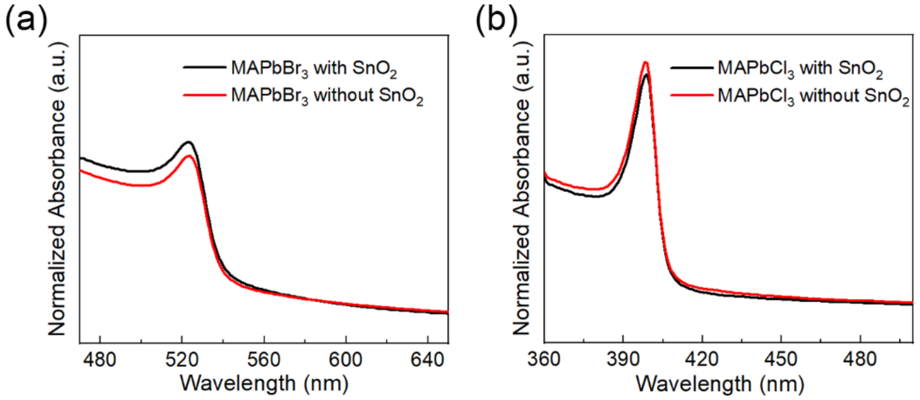

Figure S19. The UV-vis absorption spectrum of (a) $MAPbBr_3$ film and (b) $MAPbCl_3$ film with and without SnO_2 interlayer.

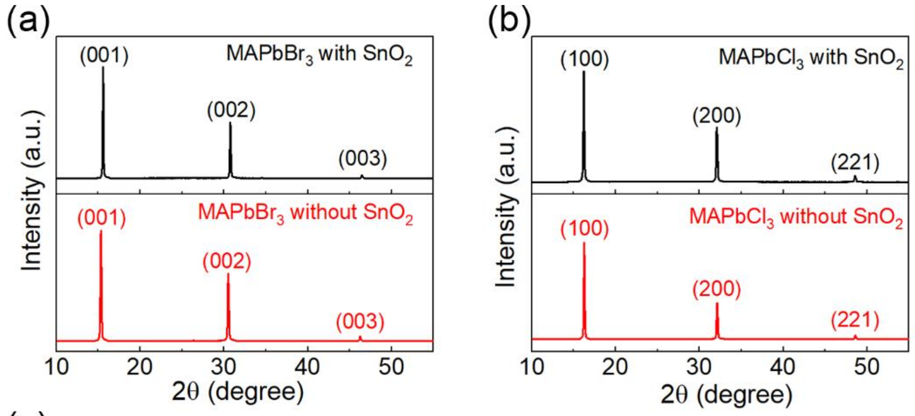

(c)

	d (Å)	FWHM (°)
$MAPbBr_3$ without SnO_2	5.778	0.158
$MAPbBr_3$ with SnO_2	5.683	0.123
$MAPbCl_3$ without SnO_2	5.447	0.132
$MAPbCl_3$ with SnO_2	5.461	0.154

Figure S20. The XRD pattern of the (a) $MAPbBr_3$ and (b) $MAPbCl_3$ films with and without SnO_2 interlayer. The lattice parameters are shown in the (c) table, which show the SnO_2 interlayer does not cause regular changes in the structure of perovskite films.
